# Exercise promotes skeletal muscle growth in adolescents via modulating Mettl3-mediated m6A methylation of MyoD in muscle satellite cells

**DOI:** 10.1186/s11658-024-00670-x

**Published:** 2024-12-04

**Authors:** Shujing Feng, Hao Zhou, Xingzuan Lin, Siyuan Zhu, Huifang Chen, Han Zhou, Ru Wang, Peng Wang, Xiexiang Shao, Jianhua Wang

**Affiliations:** 1 Xinhua Hospital affiliated to Shanghai Jiao Tong University School of Medicine, Shanghai, China; 2 School of Exercise and Health, Shanghai University of Sport, Shanghai, China; 3 Department of Sports Medicine, Peking University Third Hospital, Institute of Sports Medicine of Peking University, Beijing, China; 4 Department of Hand Surgery, Huashan Hospital, Fudan University, Shanghai, China

**Keywords:** Exercise, Adolescent skeletal muscle growth, MuSCs, Mettl3, m6A methylation, Betaine

## Abstract

**Background:**

Exercise exerts positive impacts on skeletal muscle health and homeostasis. Emerging evidence suggests that m6A methylation is involved in various physiological processes. However, the impact of exercise on adolescent skeletal muscle growth and the underlying epigenetic mechanisms remain poorly understood.

**Methods:**

The lower-limb skeletal muscles were harvested from exercise and control groups to compare the skeletal muscle growth in adolescents. mRNA sequencing was conducted to explore the mechanisms underlying enhanced skeletal muscle growth following exercise. The effects and mechanisms of Mettl3-mediated m6A methylation on adolescent skeletal muscle growth were investigated using muscle satellite cell (MuSC)-specific *Mettl3* knockout (KO) mice. The potential function of MyoD for skeletal muscle growth in adolescents was explored by phenotypes after overexpression and evaluation of in vivo myogenesis. Additionally, the effects of the methyl donor betaine on adolescent skeletal muscle growth were investigated in vitro and in vivo.

**Results:**

Exercise could promote skeletal muscle growth in adolescents. Sequencing data analysis and confirmation assays uncovered that exercise significantly increased Mettl3-mediated m6A methylation and elevated the expression levels of activation marker MyoD in MuSCs. Establishment of MuSC-specific *Mettl3* KO mice further demonstrated that Mettl3-mediated m6A methylation in MyoD contributed to skeletal muscle growth during adolescence. Mettl3-mediated m6A methylation regulated MyoD mRNA stability at the posttranscriptional level in MuSCs, with a functional site at 234 bp A. Increased expression of MyoD could contribute to myogenesis of adolescent MuSCs. Furthermore, the methyl donor betaine could enhance MyoD expression, contributing to MuSCs activation and skeletal muscle growth in adolescents by boosting m6A methylation levels.

**Conclusions:**

Exercise promoted skeletal muscle growth in adolescents through facilitating *MyoD* mRNA stability of MuSCs in a Mettl3-mediated m6A-dependent manner. The methyl donor betaine could be a potential alternative to exercise for promoting adolescent skeletal muscle growth by directly augmenting the global levels of m6A methylation. These findings may provide a theoretical foundation for encouraging daily fitness exercise and ensuring healthy growth in adolescents.

**Supplementary Information:**

The online version contains supplementary material available at 10.1186/s11658-024-00670-x.

## Introduction

Acting as the body’s largest motor organ, skeletal muscle is crucial for facilitating physical activities and sustaining overall health [1]. Puberty is accompanied by rapid skeletal muscle growth, a process that requires participation of muscle satellite cells (MuSCs) [2–4]. During this period, activated MuSCs (PAX7^+^/MyoD^+^) embark on their myogenic program, entering the cell cycle, proliferating, differentiating, and fusing, which eventually leads to an increase in the myofiber cross-sectional area [5]. Additionally, a subset of activated MuSCs engage in self-renewal and return to the niche to establish the MuSC pool [6]. MyoD serves as a marker protein for activation of MuSCs and determines the initiation of the myogenic program in MuSCs [7, 8]. Thus, MyoD expression in MuSCs is vital for pubertal skeletal muscle growth.

Exercise can effectively enhance adult skeletal muscle performance, counteract sarcopenia, and ameliorate age-related muscular degeneration in the elderly by regulating MuSCs [9–12]. Investigating the intricate mechanisms underlying these phenomena is of paramount importance. It has been reported that exercise is able to protect activated MuSCs from exhaustion by activating the Igfbp7–Akt–mTOR axis [9]. Voluntary wheel running has been shown to upregulate MyoD expression in aged MuSCs, correlating with the activation of Wnt/β-catenin signaling [13]. However, there is a lack of research clarifying the role of exercise in adolescent skeletal muscle growth and the underlying mechanisms.

m6A methylation, one of the most highly dynamic and reversible epigenetic modifications, stands out as the most prevalent internal cotranscriptional modification in eukaryotic RNAs [14]. Its involvement spans various pathophysiological processes, including mammalian development [15], tumor progression [16], and cell fate determination [17]. Principally, methyltransferases such as METTL3, METTL14, and WTAP, are responsible for catalyzing m6A methylation of adenosine on mRNA molecules [18]. Previous research has demonstrated the preventive or therapeutic effects of exercise by modulating m6A methylation levels in tissues such as the brain or cardiovascular system [19–21]. Recent studies have also identified that Mettl3-mediated m6A methylation regulated the differentiation process in mouse C2C12 myoblasts [22, 23]. However, it remains unknown whether exercise could promote adolescent skeletal muscle growth by regulating m6A methylation in MuSCs.

In this study, we established exercise mouse models to investigate the impact of exercise on skeletal muscle growth during adolescence, as well as the underlying epigenetic modification mechanisms. Here, our findings revealed that exercise during adolescence first contributed to the upregulation of Mettl3 in MuSCs, which further increased the expression of the activation marker MyoD through m6A methylation. This process was achieved by maintaining the stability of *MyoD* mRNA with a functional site at 234 bp A. The increased activation of MuSCs promoted skeletal muscle growth in adolescents. Additionally, the methyl donor betaine could be a potential alternative to exercise for promoting adolescent skeletal muscle growth by directly increasing global levels of m6A methylation.

## Materials and methods

### Animals and exercise model

Animal experimentation protocols were approved by the local institution’s ethical committee (approval no. XHEC-F-2024-042). Wild-type C57BL/6J male mice aged 3 weeks were purchased from GemPharmatech (Nanjing, China). *Pax7-CreERT2* mice (011763) were procured from Jackson Laboratory, while *Mettl3*^*f/f*^ mice (strain no. T006659) were obtained from GemPharmatech (Nanjing, China). MuSC-specific Mettl3 conditional knockout mice were induced through intraperitoneal administration of 100 µl of 10 mg/ml tamoxifen every 2 days from 2 to 3 weeks of age. The mice were allowed ad libitum access to food and water under a 12-h light/dark cycle. The exercise model was established in accordance with a modified protocol as described previously [24, 25]. Briefly, mice underwent a familiarization phase on the treadmill for 5 days, and the speed was gradually increased from 8 m/min to 12 m/min, for 30 min daily. Subsequently, the mice were subjected to 4 weeks of treadmill training at a speed of 12 m/min for 45 min/day, 5 days/week.

### Isolation of MuSCs

The lower-limb skeletal muscles were obtained and digested following established methodologies [26–28]. Briefly, the fresh skeletal muscle was first finely minced to a meat-paste consistency, digested for 1 h using collagenase II (Worthington Biochemical, 700–800 U/mL), followed by an additional 30 min with a mixture of collagenase II and dispase (11 U/mL). The resultant mixture underwent ten passes through a 20 gauge needle, followed by filtration through a 40 µm cell strainer (Corning). To isolate MuSCs, the cell suspension was incubated with following antibodies for 45 min at 4 °C: a cocktail of APC anti-CD45, APC anti-CD31, Biotin anti-VCAM1, and FITC anti-Sca1. Subsequently, PE/Cy7 streptavidin was used for staining with an additional 15 min. Finally, using a BD Influx sorter, the CD31^−^/CD45^−^/Scal1^−^/VCAM1^+^ MuSCs were isolated by fluorescence-activated cell sorting (FACS).

### EdU assay

During the final week of the exercise experiment, EdU (Yeasen, 40284ES76, 5 mg/kg body weight) was administered intraperitoneally daily to mice. Twelve hours later, MuSCs were isolated using FACS. The freshly isolated MuSCs were subsequently plated and fixed in 4% paraformaldehyde for further experiments.

### Cell culture and differentiation

In collagen-coated dishes, primary MuSCs were cultured in F10 basal medium supplemented with 20% FBS, 5 ng/mL IL-13, 5 ng/mL TNF-α, 5 ng/mL IFN-γ, 5 ng/mL IL-1α, 2.5 ng/mL bFGF, and 1% penicillin–streptomycin under optimal conditions of 37 ℃ with 5% CO_2_, in line with previous literature [26]. The differentiation medium utilized was Dulbecco’s modified Eagle medium (DMEM) supplemented with 2% horse serum and 1% penicillin–streptomycin.

### Immunofluorescence staining

Cryosections or cultured cells were fixed in PBS containing 4% paraformaldehyde (Merck, 158,127) for 15 min, permeabilized in 0.5% Triton X-100 for 15 min at room temperature, and then blocked with 1% BSA (Sigma-Aldrich, 9048–46-8) in PBS for 1 h. Subsequently, the samples were incubated overnight at 4 °C with anti-Pax7 (Thermo Fisher Scientific, PA1-117, 1:100), anti-MyoD (Santa Cruz Biotechnology, sc377460, 1:200), anti-MyoD (Santa Cruz Biotechnology, sc760, 1:200), anti-MyoG ( Santa Cruz Biotechnology, sc-12732, 1:200), anti-Laminin (Abcam, ab11575, 1:500), anti-Mettl3 (Abcam, ab195352, 1:1000), and anti-MyHC (Thermo Fisher Scientific, MA5-35613, 1:1000). Following this, secondary antibodies were used to incubate the samples for 1 h. Nuclei were subsequently stained with DAPI (Sigma-Aldrich, S7113), and antifade mounting media (Invitrogen, p36935) was applied to cover the samples. Finally, a Leica SP8 confocal microscope was employed to capture the images. Immunofluorescence staining for Laminin was used to determine the boundaries of myofibres. Immunofluorescence staining of MyHC was conducted to identify the outline of myotubes. For each sample, a minimum of five independent fields of view were randomly selected for evaluation. ImageJ was applied to quantify the cross-sectional area (CSA) of myofibers and the diameter of myotubes.

### Gene expression analysis

The EZ-press RNA Purification kit (EZBioscience) was used to extract total RNA following the manufacturer’s instructions. Reverse transcription was then performed using MuLV reverse transcriptase (NEB, M0253S). Subsequently, the products were subjected to quantitative PCR reactions with Genious 2× SYBR Green Fast qPCR mix (Abclonal, RK21205) in the CFX96 real-time PCR system. The primers used are listed in Additional file 1: Table S1.

### Overexpression

Briefly, 1 μl Lipofectamine 2000 (Thermo Fisher Scientific, 11668019) and 1 μg overexpression plasmid were mixed into 250 μl of Opti-MEM I Reduced Serum Medium (Merck, 31985062), respectively. Then the mixture was added to the cultured MuSCs for transfection and subsequent experiments were conducted at 24 or 48 h after transfection.

### Western blot

Protein samples were extracted using western and IP (Immunoprecipitation) lysis buffer (Beyotime, P0013). The protein extracts were separated by sodium dodecyl sulfate–polyacrylamide gel electrophoresis (SDS–PAGE), transferred to nitrocellulose membranes, and blocked with 5% BSA in TBST for 1 h. The membranes were then incubated overnight at 4 °C with anti-Mettl3 (Abcam, ab195352, 1:1000), anti-MyoD (Santa Cruz Biotechnology, sc377460, 1:200), or anti-GAPDH (Cell Signaling Technology, 2118S, 1:5000). Afterward, the membranes were incubated with the corresponding secondary antibodies at room temperature for 1 h. Signal detection was performed using GelDoc XR (Bio-Rad) and LumiQ ECL liquid (ShareBio, SBWB012).

### m6A dot blot assay

RNA was heated at 95 °C for 3 min to disrupt the secondary structure, followed by cooling on ice for 3 min. A source imprint was left on the nitrocellulose membranes with the rough end of a 1 mL pipette tip to facilitate spiking. Two gradients of RNA (400 ng and 800 ng) were cascaded onto the membrane, air-dried for 5 min, and then UV cross-linked for 1 h. After blocking with 5% BSA in TBS for 1 h, the membranes were incubated with anti-m6A antibody (Abcam, 151,230) overnight at 4 °C. Following three washes with TBST and 1 h of incubation with secondary antibody at room temperature, chemiluminescence was utilized for signal detection.

### Methylated RNA immunoprecipitation (MeRIP)–qPCR

MeRIP was performed with m6A RNA Methylation Fragment Enrichment kit (Epigentek, P-9018) following the manufacturer’s protocol. Initially, 20 μg of total RNA was subjected to m6A immunoprecipitation, with 1/10 of the sample reserved as the input control. To facilitate m6A RNA immunocapture, an immunocapture buffer consisting of RNA samples, m6A antibody, and affinity beads was vortexed at room temperature for 90 min. Subsequently, RNA fragmentation was achieved using a cleavage enzyme mix, followed by the addition of proteinase K and RNA purification solution to remove excess proteins and isolate m6A-containing RNA from the immunoprecipitated complex. Finally, the immunoprecipitated m6A RNA was recovered using elution buffer. The level of m6A methylation in MyoD was assessed by RT–qPCR with primers listed in Additional file 1: Table S2.

### mRNA and protein stability assay

For mRNA stability assay, actinomycin D (Act-D, MCE, HY-17559, 5 μg/mL) was used to globally inhibit mRNA transcription, and then MuSCs were harvested at time points of 0, 3, 6, 9, and 12 h. RNA extraction was then conducted for RT–qPCR analysis to evaluate RNA degradation, with GAPDH employed as the normalization reference.

To evaluate protein stability, MuSCs were treated with 100 μg/mL cycloheximide (CHX, MCE, HY-12302) and harvested at 0, 3, 6, 9, and 12 h. Subsequently, MyoD and GAPDH expression was then assessed by western blot analysis.

### Luciferase reporter assays

To validate the functional m6A methylation site in MyoD, either the wild-type or mutant CDS (Coding Sequence)of MyoD was inserted behind the F-luc coding region of the pmiRGLO vector (Miaoling Biology, P0198). *Mettl3*^*f/f*^ MuSCs and *Mettl3* KO MuSCs were transfected with pmiRGLO, pmiRGLO-MyoD-CDS-WT, pmiRGLO-MyoD-CDS-Mut1 (A to G mutation at position 228), or pmiRGLO-MyoD-CDS-Mut2 (A to G mutation at position 234) for 24 h. Subsequently, luciferase activity was quantified using the Dual Luciferase Reporter Assay kit (Yeasen Biotechnology, 11402ES60). Renilla luciferase (R-luc) was used to normalize the firefly luciferase (F-luc) activity.

### Bulk RNA sequencing and analysis

The NEBNext Ultra RNA Library Prep kit from Illumina (New England Biolabs) was utilized to create RNA sequencing libraries. Subsequently, paired-end sequencing was executed on the NovaSeq X Plus sequencer with a 2× 150 bp read length. To ensure data quality, quality control procedures were undertaken on the raw paired-end reads through the applications of SeqPrep and Sickle. Differential expression analysis was conducted using DEGseq, with genes identified as significantly differentially expressed if the fold change was greater than 1.5 and the adjusted *p*-value was < 0.001 [29]. Furthermore, for deeper insights, Gene Ontology (GO) analysis was conducted using Goatools [30].

### m6A sequencing (m6A-seq) data analysis

MeRIP sequencing raw data were obtained from the Gene Expression Omnibus (GEO) repository (accession no: GSE169432). Raw sequencing reads were first mapped to the reference mouse genome (mm10) using Hisat2 software. The mapped reads from the IP and input libraries were then analyzed using the R package exomePeak to identify significant m6A peaks and differential peaks, with a significance threshold of FDR (False Discovery Rate) ≤ 0.05. The IGV software was used for visualization.

### In vivo muscle force analysis

In vivo muscle force analysis of tibialis anterior (TA) muscle was conducted using a 3-in-1 whole-animal system (Aurora Scientific; 1300A) [25]. After anesthetizing the mouse, the hind limbs were shaved and securely placed in a frame to ensure stability without hindering blood flow. Bread silk suture was used to ligate the distal TA muscle around the patellar ligament. An incision was made to expose the sciatic nerve, which was then tied off at its proximal end. The distal TA tendon suture loop was fastened to the lever arm hook of the instrument for measuring twitch and tetanus force. Each treatment was repeated for three times, and data analysis was performed using DMA software (Aurora Scientific).

### Statistical analysis

The data were presented as mean ± SD from at least three independent experiments. Group comparisons were conducted employing either a two-tailed Student’s *t*-test or one-way ANOVA, with statistical analyses performed using GraphPad Prism 9 or SPSS version 26.0. A *p*-value < 0.05 was considered significant, with *p < 0.05, **p < 0.01, ***p < 0.001, and ns representing no significance.

## Results

### Exercise could promote skeletal muscle growth in adolescents

**Fig. 1:**
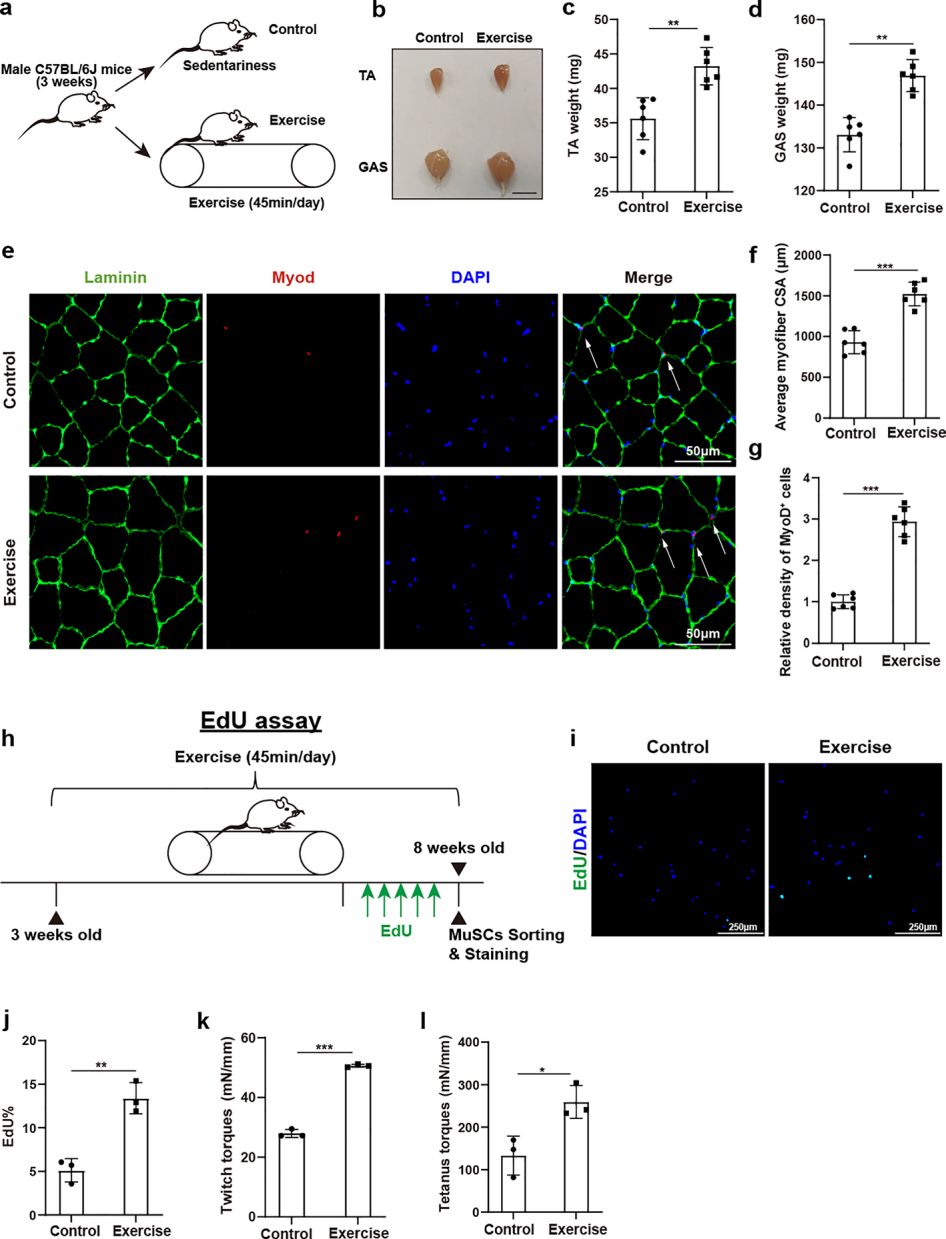
Exercise could promote skeletal muscle growth in adolescents. **a** An illustration representing the experimental design. Skeletal muscle samples were collected from both control and exercise mice following a 4 week experiment. **b** Gross appearance of tibialis anterior (TA) muscle and gastrocnemius (GAS) muscle from control and exercise mice at 8 weeks old. Scale bar, 1 cm. **c**, **d** Statistical analysis for the weight of tibialis anterior (TA) muscle and gastrocnemius (GAS) muscle from control and exercise mice at 8 weeks old (*n* = 6). **e** Immunofluorescence staining of Laminin (green) and MyoD (red) in tibialis anterior (TA) muscle from control and exercise mice at 8 weeks old. Scale bar, 50 μm. **f** Statistical analysis of the average cross-sectional area (CSA) of myofibers in tibialis anterior (TA) muscle from control and exercise mice at 8 weeks old (*n* = 6). **g** Statistical analysis of density of MyoD^+^ cells (activated MuSCs) in tibialis anterior (TA) muscle from control and exercise mice at 8 weeks old (*n* = 6). Activated MuSCs were identified as MyoD^+^/DAPI^+^ cells within the Laminin boundaries. **h** Schematic representation of the EdU assay for control and exercise mice at 8 weeks old. EdU was injected daily intraperitoneally in the last week of the exercise period. Twelve hours after the last injection, skeletal muscles were collected and MuSCs were sorted using fluorescence-activated cell sorting (FACS). **i** EdU staining (green) of freshly sorted and fixed MuSCs from control and exercise mice. Nuclei were labeled with DAPI. Scale bar, 250 μm. **j** Quantification analysis of EdU^+^ MuSCs from control and exercise mice at 8 weeks old (*n* = 3). **k**, **l** Contraction abilities (twitch and tetanus torque) of tibialis anterior (TA) muscle in control and exercise mice at 8 weeks old (*n* = 3). Data are presented as mean ± SD. *p < 0.05, **p < 0.01, ***p < 0.001, ns indicates no significant changes

To explore the impact of exercise on adolescent skeletal muscle growth, male C57BL/6J mice aged 3 weeks who underwent 4 weeks of routine exercise were assigned to the exercise group. Concurrently, sedentary age-matched mice were assigned to the control group (Fig. 1a). Initially, baseline data of tibialis anterior (TA) and gastrocnemius (GAS) muscles at 3 weeks old were collected, including gross appearance, weight, and average myofiber CSA. No discernible differences were identified in any of these parameters (Additional file 1: Fig. S1a–e). Notably, after 4 weeks of exercise, larger TA and GAS were observed in the exercise group when compared to controls (Fig. 1b). The weights of TA and GAS were significantly increased in the exercise group (Fig. 1c,d). Furthermore, immunofluorescence staining of Laminin also showed increased myofibers size of TA in the exercise group (Fig. 1e,f). Thus, exercise could increase myofiber size in adolescents. Then, we explored the potential role of MuSCs in this process. More MyoD-positive cells could be identified in muscles from the exercise group, indicating there existed more activated MuSCs after exercise (Fig. 1e,g). Furthermore, in vivo EdU staining assay was performed. EdU was injected daily intraperitoneally during the last week of the exercise period. Twelve hours after the last injection, skeletal muscles were collected, and fresh MuSCs were obtained using FACS and fixed by 4% PFA(Paraformaldehyde) (Fig. 1h). The EdU staining revealed that there were more proliferative MuSCs in the exercise group (Fig. 1i,j). Combined, these data indicated that the role of exercise for skeletal muscle growth could be ascribed to MuSC-dependent mechanisms. Furthermore, the muscular function of both the exercise and control groups was evaluated. Enhanced contraction ability of twitch and tetanus force of TA muscle could be detected in the exercise group, indicating better muscle function caused by exercise (Fig. 1k,l). Taken together, these results suggested that exercise could promote skeletal muscle growth in adolescents.

### Exercise contributed to increased expression of Mettl3 and MyoD of MuSCs in adolescents

**Fig. 2:**
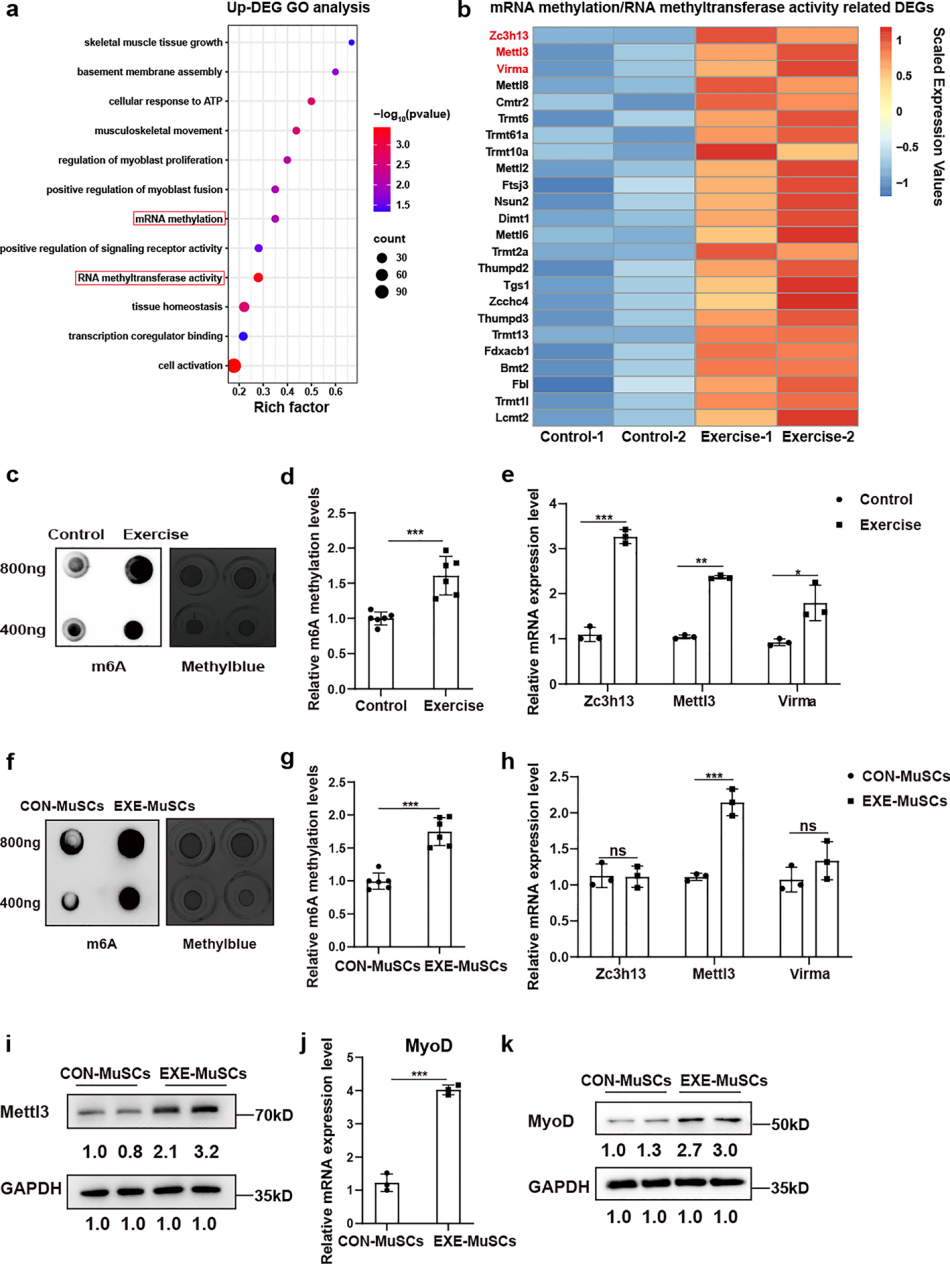
Exercise contributed to increased expression of Mettl3 and MyoD of MuSCs in adolescents. **a** Bubble chart of GO enrichment analysis of upregulated differentially expressed genes (DEGs) in tibialis anterior (TA) muscle from exercise mice when compared to those from control mice. **b** Heat map of differentially expressed genes (DEGs) associated with the GO terms of mRNA methylation and RNA methyltransferase activity between control and exercise mice. **c**, **d** Dot-blotting and quantification analysis for m6A methylation levels in tibialis anterior (TA) muscle between control and exercise mice. A twofold mass gradient of RNA samples was loaded. Methylene blue staining was used for loading controls (*n* = 3). **e** The relative mRNA expression of *Zc3h13*, *Mettl3*, and *Virma* in the tibialis anterior (TA) muscle between control and exercise mice (*n* = 3). **f**, **g** Dot-blotting and quantification analysis for m6A methylation levels of MuSCs isolated from control (CON-MuSCs) and exercise mice (EXE-MuSCs). Methylene blue staining was used as loading controls (*n* = 3). **h** The relative mRNA expression of *Zc3h13*, *Mettl3*, and *Virma* for MuSCs isolated from control (CON-MuSCs) and exercise mice (EXE-MuSCs) (*n* = 3). **i** The protein levels of Mettl3 and GAPDH for MuSCs isolated from control (CON-MuSCs) and exercise mice (EXE-MuSCs) (*n* = 3). **j** The relative mRNA expression of *MyoD* for MuSCs isolated from control (CON-MuSCs) and exercise mice (EXE-MuSCs) (*n* = 3). **k** The protein levels of MyoD and GAPDH for MuSCs isolated from control (CON-MuSCs) and exercise mice (EXE-MuSCs) (n = 3). Data are presented as mean ± SD. * *p* < 0.05, **p < 0.01, ***p < 0.001, ns indicates no significant changes

To investigate the underlying mechanisms governing the effects of exercise on adolescent skeletal muscle growth, we conducted RNA sequencing (RNA-seq) comparing TA from exercised mice to those from control mice. A total of 6972 (5873 upregulated and 1099 downregulated) differentially expressed genes (DEGs) were identified (Additional file 2: Table S1). Consistent with the aforementioned results, GO analysis of upregulated DEGs showed enriched GO terms including skeletal muscle tissue growth, regulation of myoblast proliferation, and positive regulation of myoblast fusion (Fig. 2a). Moreover, it was worth noting that GO terms including mRNA methylation and RNA methyltransferase were also enriched (Fig. 2a), indicating exercise might affect the RNA modification of adolescent skeletal muscle. In the DEGs list of these two GO terms, genes that were closely associated with m6A methylation were identified, including *Zc3h13*, *Mettl3*, and *Virma* (Fig. 2b). To validate the findings of RNA-seq, the m6A level in skeletal muscle was evaluated by dot blot assay, which revealed a substantial increase in global m6A methylation levels in adolescent mice following exercise (Fig. 2c, d). Furthermore, RT–qPCR confirmed the RNA-seq results that the expression of *Zc3h13*, *Mettl3*, and *Virma* in skeletal muscle was significantly increased in the exercise group (Fig. 2e). However, the expression levels of other methyltransferases, including *Mettl14*, *Mettl16*, and *Wtap*, did not show significant differences (Additional file 1: Fig. S2a).

Since MuSC-dependent mechanisms could play a crucial role in adolescent skeletal muscle growth after exercise, we next investigated the potential effects of exercise for m6A methylation of MuSCs. Freshly sorted MuSCs were cultured in growth medium, followed by subsequent assays. Consistently, the dot-blot assay also revealed elevated m6A methylation levels in MuSCs after exercise (Fig. 2f,g). However, RT–qPCR showed that only the expression of *Mettl3* was upregulated (Fig. 2h, Additional file 1: Fig. S2b). The increased expression of Mettl3 in MuSCs from the exercise group was further corroborated by western blot analysis and immunofluorescence staining (Fig. 2i, Additional file 1: Fig. S2c,d). In addition, since the RNA-seq data also enriched GO terms associated with activation of MuSCs, we confirmed that the key activation marker MyoD was significantly upregulated in MuSCs from the exercise group by RT–qPCR, western blot analysis, and immunofluorescence staining (Fig. 2j,k; Additional file 1: Fig. S2e,f). Collectively, these findings suggest that exercise contributed to the increased expression of Mettl3 and MyoD of MuSCs in adolescents.

### Mettl3-mediated m6A methylation is crucial for MyoD expression in MuSCs

**Fig. 3:**
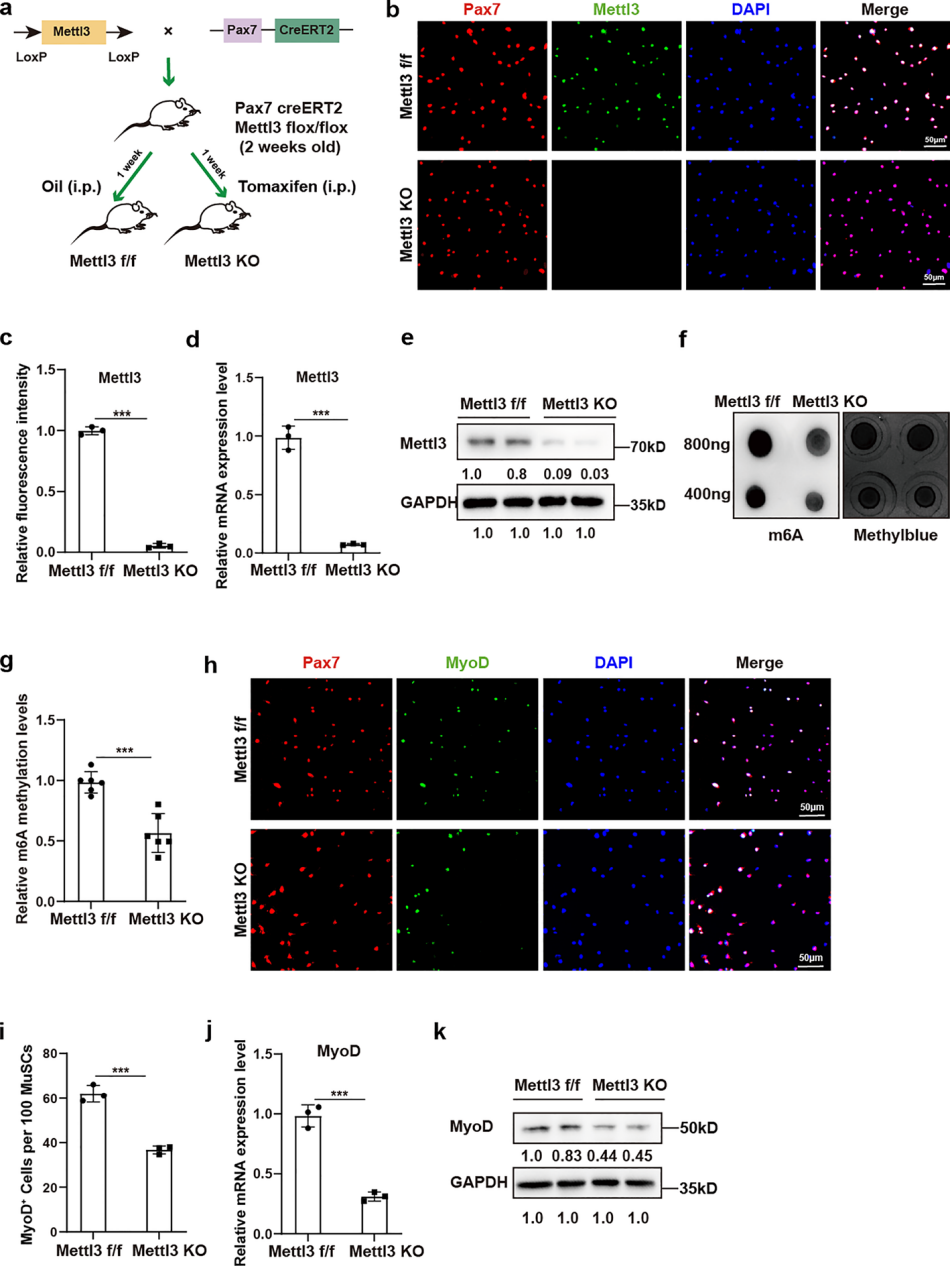
Mettl3-mediated m6A methylation is crucial for MyoD expression in MuSCs. **a** An illustration representing the generation of MuSC-specific *Mettl3* knockout (KO) mice. **b**, **c** Immunofluorescence staining of Mettl3 (green) and statistical analysis of fluorescence intensity in *Mettl3*^*f/f*^ and *Mettl3* KO MuSCs (*n* = 3). *Mettl3*^*f/f*^ and *Mettl3* KO MuSCs were respectively isolated from MuSC-specific *Mettl3*^*f/f*^ and *Mettl3* KO mice. Scale bar, 50 μm. **d** The relative mRNA expression of Mettl3 in *Mettl3*^*f/f*^ and *Mettl3* KO MuSCs (*n* = 3). **e** The protein levels of Mettl3 and GAPDH in *Mettl3*^*f/f*^ and *Mettl3* KO MuSCs (*n* = 4). **f**, **g** Dot-blotting and quantification analysis for m6A methylation levels in *Mettl3*^*f/f*^ and *Mettl3* KO MuSCs. A twofold mass gradient of RNA samples were loaded. Methylene blue staining was used for the loading controls (*n* = 3). **h**, **i** Immunofluorescence staining and qualification analysis of MyoD^+^ cells in *Mettl3*^*f/f*^ and *Mettl3* KO MuSCs (*n* = 3). Scale bar, 50 μm. **j** The relative mRNA expression of *MyoD* in *Mettl3*^*f/f*^ and *Mettl3* KO MuSCs (*n* = 4). **k** The protein levels of MyoD and GAPDH in *Mettl3*^*f/f*^ and *Mettl3* KO MuSCs (*n* = 3). Data are presented as mean ± SD. *p < 0.05, **p < 0.01, ***p < 0.001, ns indicates no significant changes

Given that m6A methylation can regulate genes posttranscriptionally, we next investigated the potential role of Mettl3-mediated m6A methylation in regulating MyoD expression in MuSCs. MuSC-specific *Mettl3* KO mice were generated by intraperitoneal injection of tamoxifen into *Pax7-CreERT2*; *Mettl3*^*f/f*^ mice every 2 days for 1 week, starting at 2 weeks of age and ending at 3 weeks of age (Fig. 3a). MuSCs were isolated and sorted following established producers [26]. Knockout of Mettl3 in MuSCs was verified by immunofluorescence staining, RT–qPCR, and western blot analysis (Fig. 3b–e). Consistently, we observed decreased global m6A methylation levels in MuSCs from MuSC-specific *Mettl3* KO mice (Fig. 3f,g). Moreover, *Mettl3* knockout resulted in a reduced number of MyoD^+^ cells and lower expression levels of MyoD, indicating impaired activation of MuSCs (Fig. 3h–k). Collectively, these data suggest that Mettl3-mediated m6A methylation is crucial for MyoD expression in MuSCs.

### Mettl3-mediated m6A methylation of MuSCs contributes to skeletal muscle growth in adolescents

**Fig. 4:**
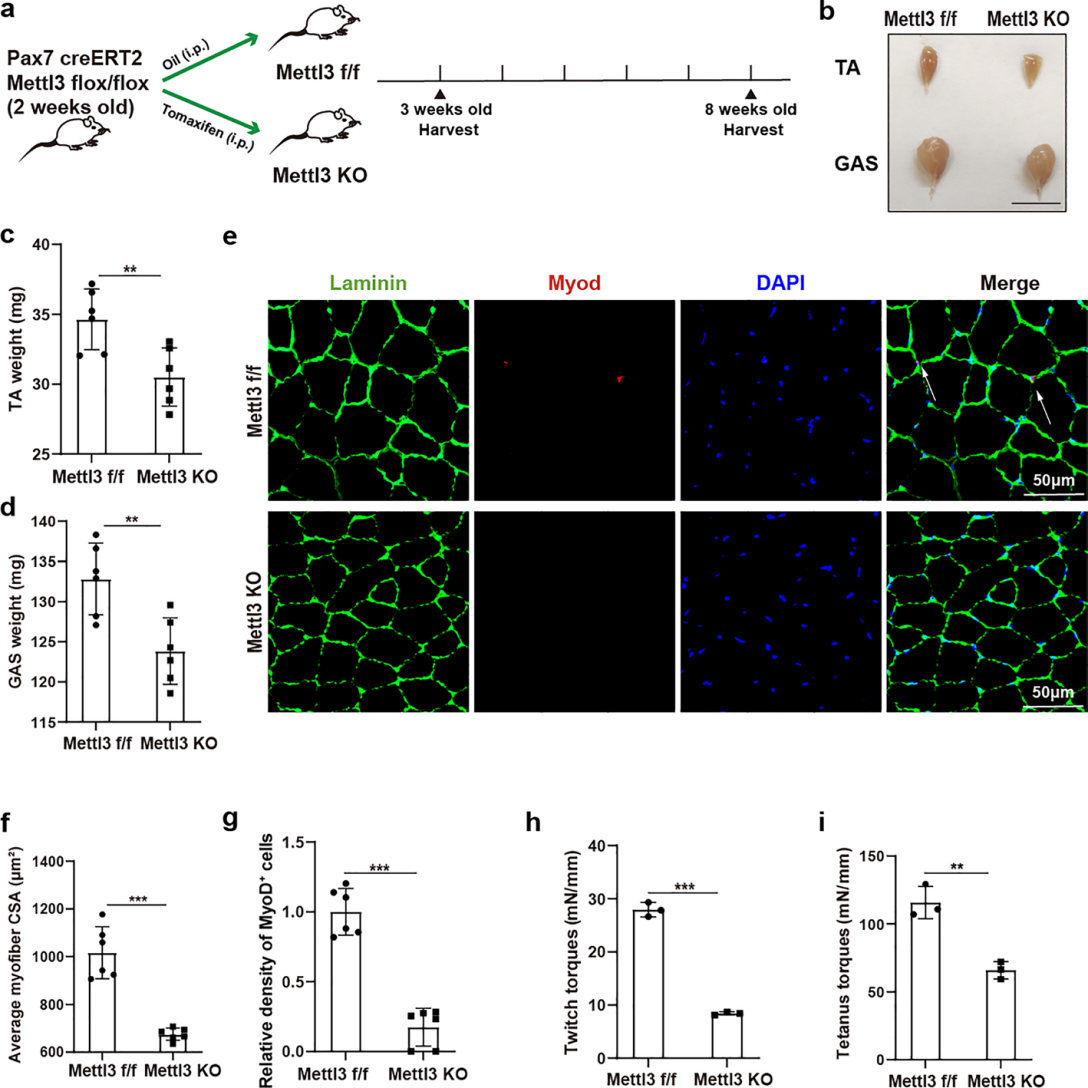
Mettl3-mediated m6A methylation of MuSCs contributes to skeletal muscle growth in adolescents. **a** Schematic representation for investigating Mettl3-mediated m6A methylation on skeletal muscle growth in adolescents. Skeletal muscle samples were collected from MuSC-specific *Mettl3*^*f/f*^ and *Mettl3* KO mice at 3 weeks old and 8 weeks old, respectively. **b** Gross appearance of tibialis anterior (TA) muscle and gastrocnemius (GAS) muscle of MuSCs specific *Mettl3*^*f/f*^ and *Mettl3* KO mice at 8 weeks old. Scale bar, 1 cm. **c**, **d** Statistical analysis for the weight of tibialis anterior (TA) muscle and gastrocnemius (GAS) muscle of MuSC-specific *Mettl3*^*f/f*^ and *Mettl3* KO mice at 8 weeks old (*n* = 6). **e** Immunofluorescence staining of Laminin (green) and MyoD (red) of tibialis anterior (TA) muscle of MuSCsspecific *Mettl3*^*f/f*^ and *Mettl3* KO mice at 8 weeks old (*n* = 6). Scale bar, 50 μm. **f** Statistical analysis of the average cross-sectional area (CSA) of myofibers in tibialis anterior (TA) muscle from MuSC-specific *Mettl3*^*f/f*^ and *Mettl3* KO mice at 8 weeks old (*n* = 6). **g** Statistical analysis of density of MyoD^+^ cells (activated MuSCs) in tibialis anterior (TA) muscle from MuSC-specific *Mettl3*^*f/f*^ and *Mettl3* KO mice at 8 weeks old (*n* = 6). **h**, **i** Contraction abilities (twitch and tetanus torque) of tibialis anterior (TA) muscle in MuSC-specific *Mettl3*^*f/f*^ and *Mettl3* KO mice at 8 weeks old (*n *= 3). Data are presented as mean ± SD. *p < 0.05, **p < 0.01, ***p < 0.001, ns indicates no significant changes

The function of Mettl3-mediated m6A methylation of MuSCs for adolescent skeletal muscle growth was further investigated. Firstly, 2-week-old *Pax7-CreERT2*; *Mettl3*^*f/f*^ mice received intraperitoneal injections of tamoxifen (*Mettl3* KO) or oil vehicle (*Mettl3*^*f/f*^) every other day for 1 week. Skeletal muscles from MuSCs specific *Mettl3* KO and *Mettl3*^*f/f*^ mice were harvested at 3 weeks and 8 weeks of age, respectively (Fig. 4a). The MuSC- specific *Mettl3* KO mice exhibited significantly smaller skeletal muscle size and lower skeletal muscle weight at 8 weeks old (Fig. 4b–d), although there was no difference in these parameters at 3 weeks of age (Additional file 1: Fig. S3a–c). Immunofluorescence staining analysis of TA further revealed significantly smaller myofibers size in MuSC-specific *Mettl3* KO mice at 8 weeks of age (Fig. 4e,f), while no differences were observed at 3 weeks of age (Additional file 1: Fig. S3d,e). We also conducted an analysis of activated MuSCs in MuSC specific *Mettl3* KO and *Mettl3*^*f/f*^ mice aged 8 weeks in vivo. The immunofluorescence staining analysis of MyoD indicated that Mettl3 KO significantly decreased activation of MuSCs in vivo (Fig. 4e,g). Furthermore, MuSC-specific *Mettl3* KO mice also exhibited impaired muscle function, characterized by reduced twitch and tetanus force of TA (Fig. 4h,i). Taken together, these findings suggested that Mettl3-mediated m6A methylation of MuSCs contributes to increased expression of MyoD, and thus promotes skeletal muscle growth in adolescents.

### Mettl3-mediated m6A methylation regulates the stability of *MyoD* mRNA at the posttranscriptional level in MuSCs

**Fig. 5:**
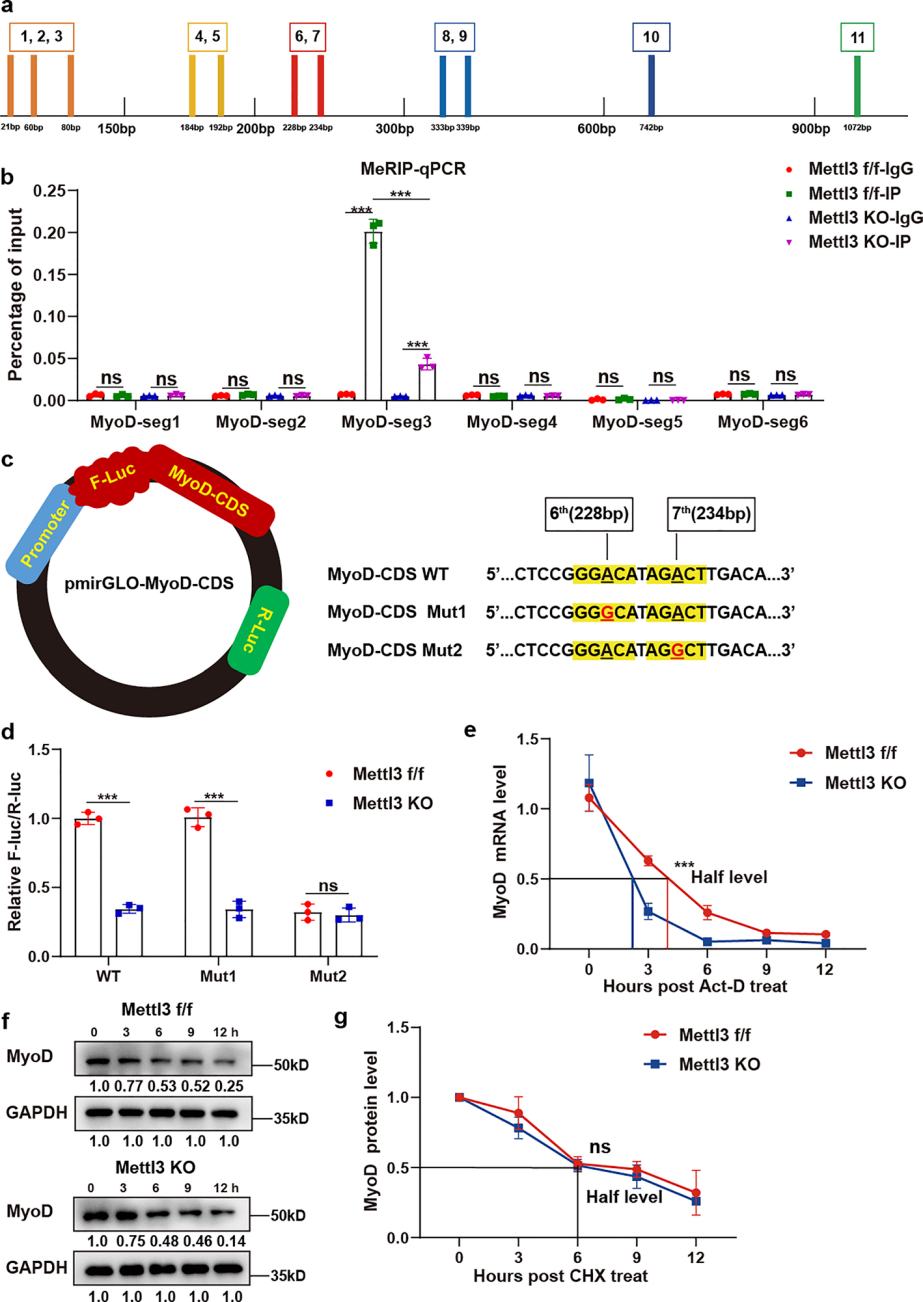
Mettl3-mediated m6A methylation regulated the stability of MyoD mRNA at the posttranscriptional level in MuSCs. **a** Schematic representation of potential m6A methylation sites in MyoD mRNA predicted by SRAMP. The MyoD mRNA sequence was divided into six segments according to the potential m6A methylation sites, encompassing solely those with high-confidence sites and very high-confidence sites. **b** MeRIP–qPCR results of six segments in MyoD. MeRIP–qPCR analysis was performed to detect the m6A enrichment of six segments between the anti-IgG group and anti-m6A group in *Mettl3*^*f/f*^ and *Mettl3* KO MuSCs (*n* = 3). **c** Schematic representation of luciferase reporter assays. Wild-type (WT) MyoD-CDS, mutant of the 6th potential site (Mut1: GGAC to GGGC), or mutant of the 7th potential site (Mut2: AGAC to AGGC) MyoD-CDS was individually inserted behind the F-luc coding region in luciferase reporter. **d** The result of relative luciferase activity. The relative luciferase activity was defined as the ratio of Firefly luciferase activity to Renilla luciferase activity (*n* = 3). **e** mRNA stability assay. *Mettl3*^*f/f*^ and *Mettl3* KO MuSCs were treated with actinomycin D (Act-D), and *MyoD* mRNA expression of *Mettl3*^*f/f*^ and *Mettl3* KO MuSCs were analyzed at the indicated time (*n* = 3). **f**, **g** Protein stability assay. *Mettl3*^*f/f*^ and *Mettl3* KO MuSCs were treated with cycloheximide (CHX), and protein expression of MyoD and GAPDH were analyzed at the indicated time by western blot analysis (*n* = 3). Data are presented as mean ± SD. *p < 0.05, **p < 0.01, ***p < 0.001, ns indicates no significant changes

To characterize the detailed mechanism of how m6A methylation regulated MyoD, we first applied SRAMP (Sequence-based RNA Adenosine Methylation Site Predictor, http://www.cuilab.cn/sramp) to predict the abundance of m6A methylation loci on MyoD. A total of 21 potential loci were identified along the full length of MyoD (Additional file 1: Fig. S4a). To determine the effective m6A methylation segments within MyoD, we formulated six primer pairs to amplify discrete regions of the MyoD sequence, targeting potential sites with high and very high confidence. MyoD-seg1 contained the 1st, 2nd, and 3rd potential m6A methylation sites; MyoD-seg2 contained the 4^th^ and 5th potential m6A methylation sites; MyoD-seg3 contained the 6 and 7th potential m6A methylation sites; MyoD-seg4 contained the 8th and 9th potential m6A methylation sites; MyoD-seg5 contained the 10th potential m6A methylation site; MyoD-seg6 contained the 11th potential m6A methylation site (Fig. 5a). MeRIP–qPCR results revealed that only the MyoD-seg3 (including the 6th and 7th potential m6A methylation sites located in the CDS region of MyoD) displayed a high level of m6A methylation in *Mettl3*^*f/f*^ MuSCs, while a notable decrease in m6A methylation levels on MyoD was detected in *Mettl3* KO MuSCs (Fig. 5b). Then, the luciferase assay was performed to verify the the 6th and 7th potential m6A methylation sites (228 bp A and 234 bp A). Specifically, wild-type (WT) MyoD-CDS, mutant of the 6th potential site (Mut1: GGAC to GGGC), or mutant of the 7th potential site (Mut2: AGAC to AGGC) MyoD-CDS was individually inserted behind the F-luc coding region in luciferase reporter plasmids (Fig. 5c). After transfection with MyoD-CDS-WT or MyoD-CDS-Mut1 reporter plasmids, a significant decrease in luciferase activity in *Mettl3* KO MuSCs was found when compared to that in *Mettl3*^*f/f*^ MuSCs (Fig. 5d). This indicates that the 6th site was not the valid m6A methylation site. In contrast, there was no significant difference of luciferase activity between *Mettl3* KO and *Mettl3*^*f/f*^ cells when transfected with the MyoD-CDS-Mut2 reporter plasmid (Fig. 5d), suggesting that the 7th potential m6A methylation site of MyoD (234 bp A) functioned as the valid site governing MyoD m6A methylation levels. Furthermore, we also conducted unbiased MeRIP-seq analysis according to data of muscle stem cell-specific *Mettl3* KO and WT MuSCs obtained from GEO (GSE169432) [31]. The m6A methylation peak calling was performed by algorithm exomePeak. The results confirmed that MyoD was modified by m6A methylation, showing a significant decrease in m6A enrichment in the region of chromosome 7 from positions 46,376,530 to 46,376,749, which corresponded to gene sequence positions 57 bp to 276 bp (Additional file 1: Fig. S4b, c). Since the functional m6A modification site 234 bp A found in our study was also located in this region, m6A-seq analysis further supports our findings.

Next, we assessed the effects of Mettl3-mediated methylation on the expression of MyoD. We constructed plasmids encoding WT or mutant MyoD CDS (with a mutation at the 234 bp position where A was changed to G), and they were transfected into *Mettl3*^*f/f*^ or *Mettl3* KO MuSCs. The results showed that in *Mettl3*^*f/f*^ MuSCs, the mutation led to a significant decrease in the expression level of MyoD (Additional file 1: Fig. S4d–f). In *Mettl3* KO MuSCs, due to the absence of Mettl3 for m6A methylation, there was no significant difference in expression level of MyoD between the mutated and WT groups (Additional file 1: Fig. S4d–f). Combined, these results underscored that this m6A methylation site contributed to the expression level of MyoD.

To further investigate how Mettl3-mediated m6A methylation regulated the expression of MyoD, mRNA and protein stability assays were performed. Both *Mettl3*^*f/f*^ and *Mettl3* KO MuSCs were treated with Act-D or CHX to block global transcription or translation, respectively. The results unveiled a significantly shorter half-life of *MyoD* mRNA in *Mettl*3 KO MuSCs (Fig. 5e). However, no significant difference in the half-life of MyoD protein was observed between *Mettl3*^*f/f*^ and *Mettl3* KO MuSCs as detected by western blot analysis (Fig. 5f,g). Taken together, these findings indicated that Mettl3-mediated m6A methylation regulated *MyoD* mRNA stability at the posttranscriptional level in MuSCs.

### Increased expression of MyoD could contribute to myogenesis of adolescent MuSCs

**Fig. 6:**
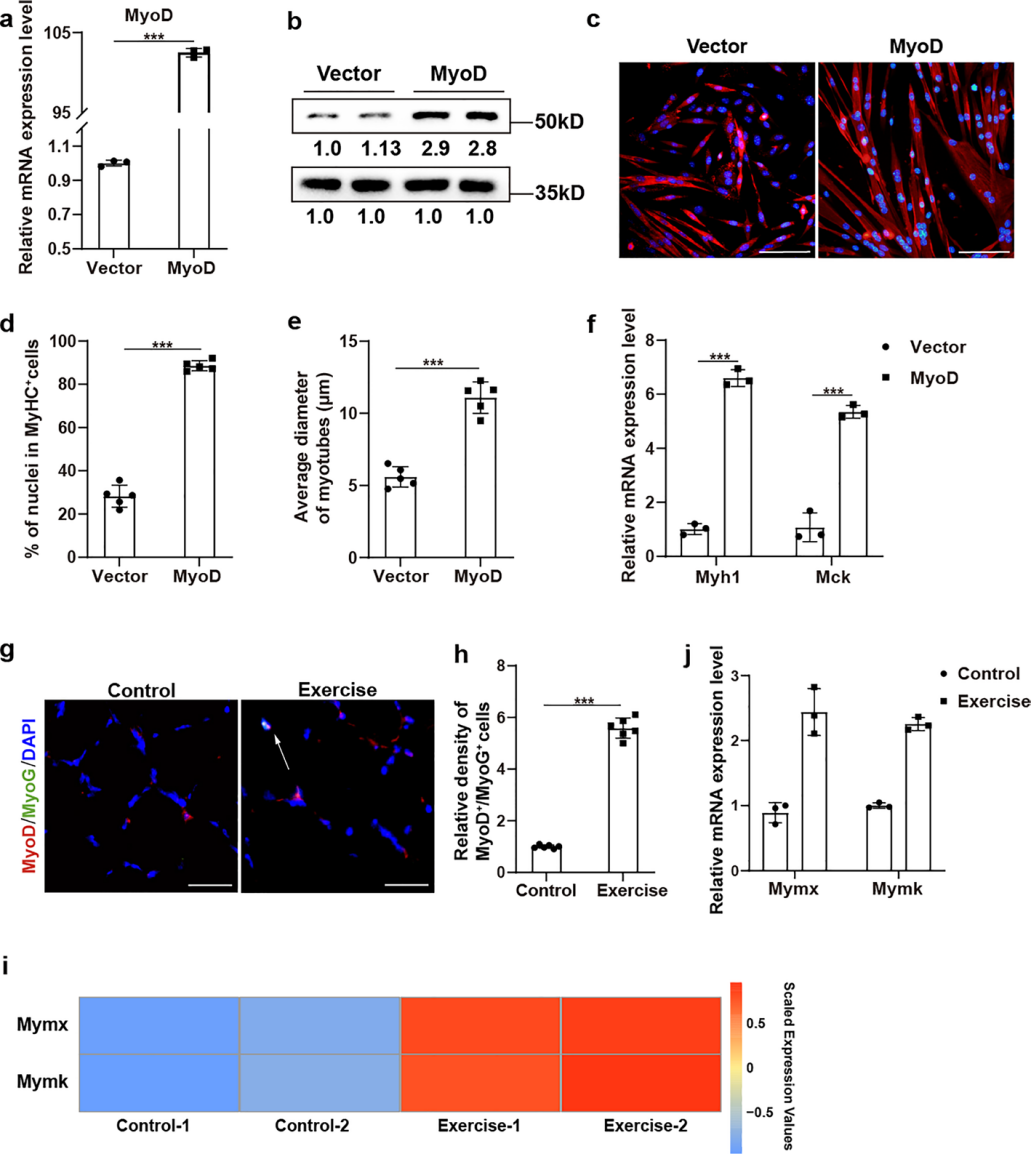
Increased expression of MyoD could contribute to myogenesis of adolescent MuSCs. **a** The relative mRNA expression of *MyoD* in adolescent MuSCs after transfected with pcDNA-MyoD-WT (MyoD) or empty plasmid (vector) (*n* = 3). **b** The protein levels of MyoD and GAPDH in adolescent MuSCs after transfection with pcDNA-MyoD-WT (MyoD) or empty plasmid (vector) (*n* = 4). **c** Immunofluorescence staining of MyHC (red) with differentiated adolescent MuSCs. Adolescent MuSCs were differentiated for 24 h after transfection with pcDNA-MyoD-WT (MyoD) or empty plasmid (vector). Nuclei were labeled with DAPI. Scale bars, 50 μm. **d** Statistical analysis of the average diameter of myotubes from differentiated adolescent MuSCs transfected with pcDNA-MyoD-WT (MyoD) or empty plasmid (vector) (*n* = 5). **e** Statistical analysis of the differentiation efficiency for adolescent MuSCs transfected with pcDNA-MyoD-WT (MyoD) or empty plasmid (vector) (*n* = 5). **f** The relative mRNA expression of *Myh1* and *Mck* in differentiated adolescent MuSCs transfected with pcDNA-MyoD-WT (MyoD) or empty plasmid (vector) (*n* = 3). **g** Immunofluorescence staining of MyoD (red) and MyoG (green) in tibialis anterior (TA) muscle from control and exercise mice at 8 weeks old. Scale bar, 25 μm. **h** Statistical analysis of MyoD^+^/MyoG^+^ cells in tibialis anterior (TA) muscle from control and exercise mice at 8 weeks old (*n* = 6). **i** Heat map of expression of Mymx and Mymk between control and exercise mice. **j** The relative mRNA expression of *Mymx* and *Mymk* in tibialis anterior (TA) muscle from control and exercise mice at 8 weeks old (*n* = 3). Data are presented as mean ± SD. *p < 0.05, **p < 0.01, ***p < 0.001, ns indicates no significant changes

We then explore the role of increased MyoD in MuSCs for exercise-induced muscle growth. It has been widely acknowledged that MyoD is an activator of differentiation-specific genes [32, 33]. The overexpression plasmid encoding MyoD was first transfected into MuSCs from adolescent mice and overexpression of MyoD was confirmed by RT–qPCR and western blot (Fig. 6a, b). Then, myogenic induction was performed in myogenic differentiation medium for 24 h. After differentiation, a significantly increased fusion index and larger myotube diameter were identified in the MyoD overexpression group (Fig. 6c–e). In addition, the expression level of genes marking differentiation such as *Myh1* and *Mck* were significantly upregulated in the MyoD overexpression group (Fig. 6f). These data indicate that increased expression of MyoD could significantly promote the myogenic differentiation for MuSCs isolated from adolescent mice. During the myogenic differentiation program, MyoG would be activated by MyoD and subsequently play a major role in terminal myogenic differentiation [33–35]. The staining of MyoD and MyoG found that there were significantly increased MyoD^+^/MyoG^+^ myocytes in skeletal muscle after exercise (Fig. 6g,h), indicating that MuSCs with increased MyoD caused by exercise further progressed into the late myogenic differentiation program for myotube formation and skeletal muscle growth. Since the MyoD–Myomixer/Myomaker axis determines the process of myotube formation [36, 37], the potential change in Myomixer and Myomaker by exercise was evaluated. Both the RNA-seq data and RT–qPCR analysis showed significantly increased expression of these two key fusogenic regulators in adolescent skeletal muscle after exercise (Fig. 6i,j), indicating the activation of the MyoD–Myomixer/Myomaker axis caused by exercise. Thus, these data, combined with published findings, demonstrate that exercise could increase the expression of MyoD of MuSCs, and then promote the myogenic differentiation program and muscle growth for adolescents.

### Methyl donor betaine induces MuSCs activation and promotes skeletal muscle growth in adolescent mice

**Fig. 7:**
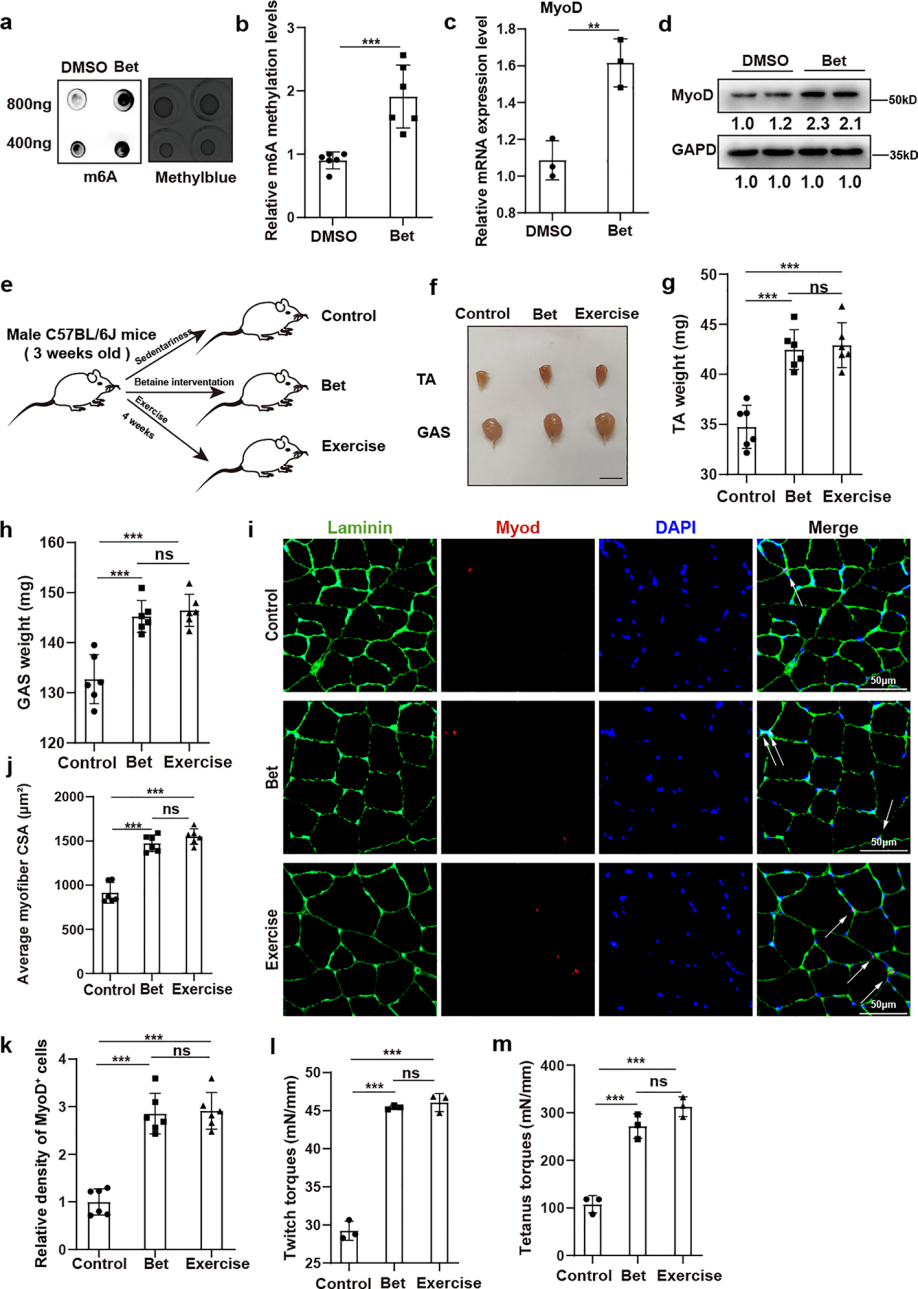
Methyl donor betaine induced MuSCs activation and promoted skeletal muscle growth in adolescent mice. **a**, **b** Dot-blotting and quantification analysis for m6A methylation levels in MuSCs with treatment of dimethyl sulfoxide (DMSO) or betaine (bet) (*n* = 3). Twofold mass gradient of RNA samples were loaded. Methylene blue staining was used as loading controls. **c** The relative mRNA expression of *MyoD* in MuSCs with treatment of DMSO (DMSO) or betaine (bet) (*n* = 3). **d** The protein levels of MyoD and GAPDH in MuSCs with treatment of DMSO (DMSO) or betaine (bet) (*n* = 4). **e** An illustration representing the experimental design. Three-week-old mice were randomly assigned to the control group (control), betaine intervention group (bet), or exercise group (exercise). Skeletal muscles from the three groups were harvested after 4 weeks. **f** Gross appearance of tibialis anterior (TA) muscle and gastrocnemius (GAS) muscle in control group (control), betaine intervention group (bet), and exercise group (exercise). Scale bar, 1 cm. **g**, **h** Statistical analysis for the weight of tibialis anterior (TA) muscle and gastrocnemius (GAS) muscle in control group (control), betaine intervention group (bet), and exercise group (exercise) (*n* = 6).** i** Immunofluorescence staining of Laminin (green) and MyoD (red) of the tibialis anterior (TA) muscle in control group (control), betaine intervention group (bet) and exercise group (exercise). Scare bar, 50 μm. j Statistical analysis for the average cross-sectional area (CSA) of myofibers in tibialis anterior (TA) muscle from control group (control), betaine intervention group (bet) and exercise group (exercise) (*n* = 6). **k** Statistical analysis of density of MyoD^+^ cells (activated MuSCs) in tibialis anterior (TA) muscle from control group (control), betaine intervention group (bet), and exercise group (exercise) (*n* = 6). **l**, **m** Contraction abilities (twitch and tetanus torque) of tibialis anterior (TA) muscle from control group (control), betaine intervention group (bet), and exercise group (exercise) (*n* = 3). Data are presented as mean ± SD. *p < 0.05, **p < 0.01, ***p < 0.001, ns indicates no significant changes.

It has been reported that the methyl donor betaine could positively regulate m6A methylation levels and enhance myogenic differentiation in murine myoblasts [38]. Considering the significant role of m6A methylation for MuSCs discovered in the current study, 0.2 M betaine was added to the growth medium of MuSCs to explore its effect. m6A dot-blot analysis revealed increased levels of m6A methylation in MuSCs after treatment with betaine (Fig. 7a,b). Additionally, betaine treatment contributed to the elevated expression of MyoD in MuSCs, which was confirmed by RT–qPCR and western blot analysis (Fig. 7c,d). These data indicate the potential positive effects of betaine on MuSCs in vitro.

We then investigated the effects of betaine on adolescent skeletal muscle growth in vivo. Mice aged 3 weeks were randomly assigned to three groups: control group (control), betaine intervention group (bet), or exercise group (exercise) (Fig. 7e). The betaine group referred to mice receiving drinking water supplemented with 2% betaine (w/v) over a period of 4 weeks. Mice in the exercise group underwent treadmill training for 45 min at a speed of 12 m/min each day, also spanning 4 weeks, while control mice remained sedentary with regular water. After 4 weeks, the betaine and exercise groups displayed enlarged gross morphology of TA and GAS muscles compared to the control group (Fig. 7f). Furthermore, the weight of TA and GAS muscles markedly increased in both the betaine and exercise groups (Fig. 7g,h). Consistently, larger myofibers size were also observed in both the betaine and exercise groups via immunofluorescence staining of Laminin in TA (Fig. 7i,j). Immunofluorescence staining of MyoD further demonstrated more activated MuSCs in both the betaine-treated and exercise mice in vivo (Fig. 7i,k). Furthermore, improved muscle function, as indicated by enhanced contraction ability, was also found in both the betaine and exercise mice (Fig. 7l,m). It was worth noting that the beneficial effects of betaine intervention on adolescent skeletal muscle growth were comparable to exercise. These results highlight the potential of the methyl donor betaine as an effective nutritional supplement and a viable alternative to exercise for improving adolescent skeletal muscle growth.

## Discussion

**Fig. 8:**
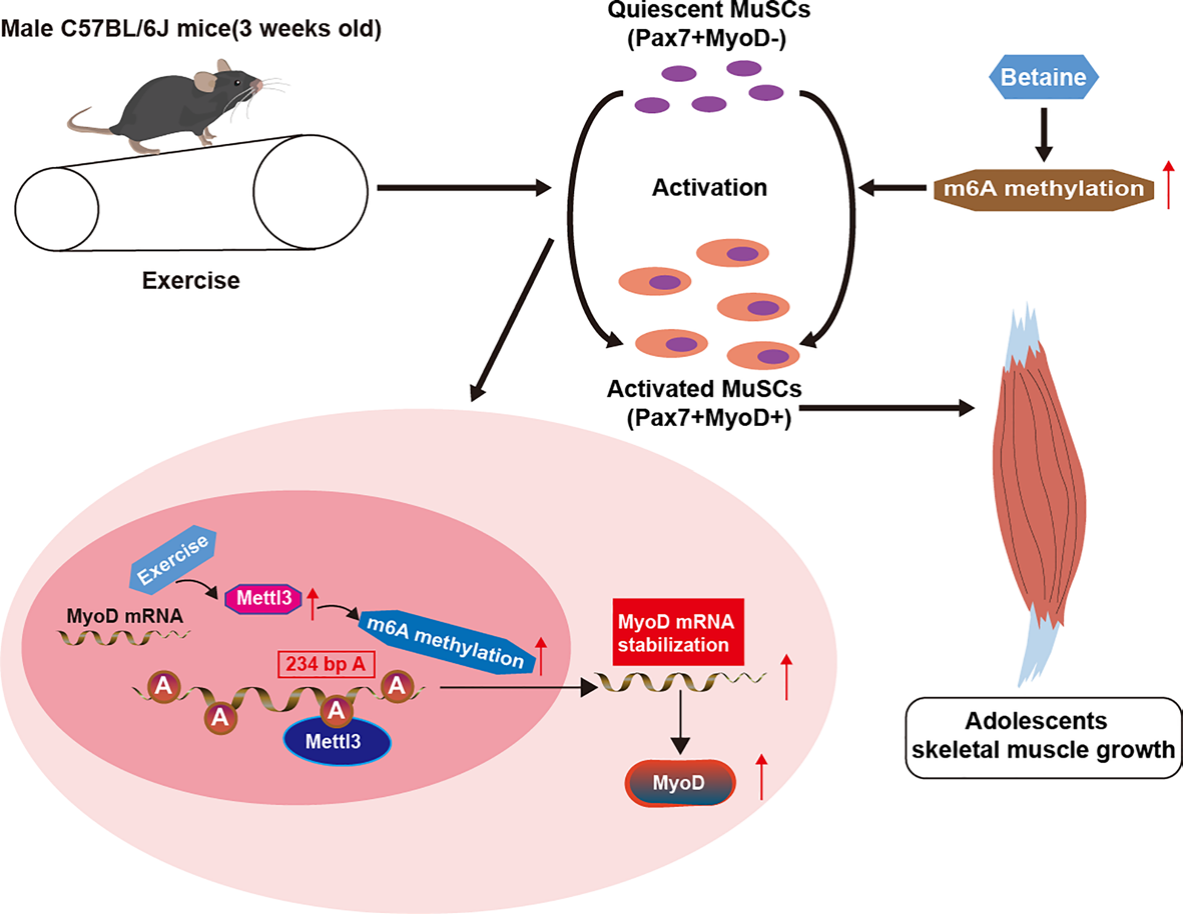
Schematic diagram of the current study. Exercise in adolescents first contributed to the upregulation of Mettl3 in MuSCs, which further increased the expression level of the activation marker MyoD by m6A methylation. This process was achieved by maintaining the stability of *MyoD* mRNA with a functional site at 234 bp A. The increased activation of pubertal MuSCs promoted adolescent skeletal muscle growth. Additionally, the methyl donor betaine could be a potential alternative to exercise in promoting adolescent skeletal muscle growth by directly augmenting the global levels of m6A methylation

Our study demonstrated that exercise during adolescence first contributed to upregulation of Mettl3 in MuSCs, which further increased the expression level of the activation marker MyoD through m6A methylation. This process was achieved by maintaining the stability of MyoD mRNA with a functional site at 234 bp A. The increased activation of MuSCs promoted skeletal muscle growth in adolescents. Additionally, the methyl donor betaine could be a substitute for exercise to promote adolescent skeletal muscle growth by directly increasing the global levels of m6A methylation (Fig. 8).

It has been reported that several environmental factors affect adolescent skeletal muscle growth, while physical activity could be one of the key contributors [39–41]. Appropriate physical activity improves skeletal muscle fitness in adolescents, encompassing both muscular strength and endurance [42]. Hao et al. demonstrated that physical activity was positively correlated with adolescent skeletal muscle mass index [43]. Yoshitake Cho et al. highlighted the potential of endurance exercise training to reshape adolescent skeletal muscle, alter the metabolic and contractile characteristics, and heighten mitochondrial oxidative capacity [44]. Adequate skeletal muscle growth during adolescence could exhibit long-term effects on body composition, greatly contributing to overall quality of life [39]. However, studies have revealed that approximately 80% of school-going adolescents failed to meet recommended physical activity levels, largely attributed to the ubiquitous use of electronic devices and a lack of exercise motivation [45, 46]. Insufficient physical activity may impair skeletal muscle growth, consequently increasing the risk of metabolic disorders and cardiovascular diseases among adolescents [47, 48]. Therefore, it is necessary to focus on skeletal muscle health and explore effective strategies to foster skeletal muscle growth in adolescents.

It has been shown that MuSCs are indispensable for skeletal muscle growth during adolescence [3]. The transition from quiescence to activation in MuSCs and the subsequent formation of myofibers represent pivotal steps in myogenesis, with MyoD playing a crucial role [7, 49–51]. Exercise serves as a common mechanical stimulus to investigate the effects on skeletal muscle homeostasis and the associated fundamental molecular mechanisms. There is evidence suggesting that exercise can effectively activate MuSCs in adults and the elderly. For instance, Chen et al. uncovered the positive impact of exercise on physiological muscle hypertrophy in adults by modulating mitochondrial metabolism in MuSCs [9]. Moreover, exercise can mitigate the adverse consequences of sarcopenia in the elderly by reactivating MuSCs [13]. Nevertheless, the influence of exercise on adolescent skeletal muscle remains unclear, and the associated cells and molecular mechanisms need to be comprehensively elucidated.

An essential finding of this study is that exercise could promote skeletal muscle growth in adolescents by increasing Mettl3-mediated m6A methylation of MyoD. Several studies have examined the role of m6A methylation in the proliferation and differentiation of primary MuSCs and C2C12 myoblasts [22, 52, 53]. Although a previous study indicated that there could be m6A modification sites in MyoD mRNA of C2C12 myoblasts [23], results from some other investigations seem contradictory [22, 53]. Qiao et al. emphasized the importance of the m6A reader YTHDC1 in MuSC activation and proliferation, showing that YTHDC1 regulated mRNA splicing and nuclear export through an m6A-dependent manner [53]. However, MyoD was not identified as a target of m6A-YTHDC1, possibly due to the diversity of other m6A readers such as YTHDF1, YTHDF2, and YTHDC2 [54, 55]. YTHDC1 might play a limited role in recognizing m6A-modified MyoD. Additionally, Ghelle’s team analyzed m6A-modified transcripts during C2C12 myoblasts proliferation and early differentiation [22]. They found no changes in the m6A methylation levels of transcription factors such as MyoD, which seems to be in conflict with our observation. This discrepancy might be due to differences in cell models or cellular states used in the studies. Generally, these data underscore the significance and complexity of the m6A regulatory network in muscle cells, emphasizing the need for further exploration to fully understand its implications.

The aforementioned outcomes indicated the potential to enhance skeletal muscle growth during puberty by modulating the m6A methylation of MyoD. Betaine (*N*,*N*,*N*-trimethylglycine) is a quaternary ammonium compound commonly found in various plants and animals [56]. One of the primary functions of betaine is to act as a methyl donor and participate in the methionine–homocysteine cycle, leading to elevated SAM levels [57]. A recent study has indicated that betaine could enhance m6A methylation levels in a dose-dependent manner [38]. Therefore, betaine was selected to investigate its impacts on MuSCs activation and skeletal muscle growth in adolescents. As expected, the current study revealed that betaine supplementation led to increased MuSC activation and promoted skeletal muscle growth in adolescents, comparable to the effects of exercise. Given the various obstacles that hinder today’s adolescents from participating in daily exercise, betaine may serve as a potential alternative to exercise from nutritional perspective.

## Conclusions

Exercise promoted skeletal muscle growth in adolescents through facilitating *MyoD* mRNA stability of MuSCs in a Mettl3-mediated m6A dependent manner. The methyl donor betaine could be a potential alternative to exercise for promoting adolescent skeletal muscle growth by directly augmenting the global levels of m6A methylation. These findings may provide a theoretical foundation for encouraging daily fitness exercise and ensuring healthy growth in adolescents.

## Supplementary Information

Supplementary Material 1.

Supplementary Material 2.

## Data Availability

The data for this study can be acquired upon reasonable request from the corresponding author. Bulk RNA sequencing data have been deposited in GEO under the accession number GSE278365.

## Abbreviations

MuSCs: Muscle satellite cells
KO: Knockout
FACS: Fluorescence-activated cell sorting
Bet: Betaine
CSA: Cross-sectional area
IP: Immunoprecipitation
MeRIP: Methylated RNA immunoprecipitation
FDR: False Discovery Rate
TA: Tibialis anterior
GAS: Gastrocnemius
CDS: Coding Sequence
Act-D: Actinomycin D
CHX: Cycloheximide
F-luc: Firefly luciferase
R-luc: Renilla luciferase
PFA: Paraformaldehyde
SRAMP: Sequence-based RNA Adenosine Methylation Site Predictor
