# Supplementary material for: Exercise promotes skeletal muscle growth in adolescents via modulating Mettl3-mediated m6A methylation of MyoD in muscle satellite cells
Source: Cell Mol Biol Lett. 2024 Dec 4;29:150. doi: 10.1186/s11658-024-00670-x (PMC11616192; doi:10.1186/s11658-024-00670-x)
Supplement: Supplementary file 1 — Supplementary Material 1. [file 11658_2024_670_MOESM1_ESM.doc]

Exercise promotes skeletal muscle growth in adolescents via modulating Mettl3-mediated m6A methylation of MyoD in muscle satellite cells

Shujing Feng ^1, †^, Hao Zhou ^2, †^, Xingzuan Lin ^3, 2, †^, Siyuan Zhu ^4, †^, Huifang Chen ^1^, Han Zhou ^2^, Peng Wang ^2*^, Xiexiang Shao ^2*^, Jianhua Wang ^1, 2*^

Data Supplement

1. Table S1
2. Table S2
3. Figure S1
4. Figure S2
5. Figure S3
6. Figure S4

| Table S1. Primer sequences for RT-qPCR | |
| --- | --- |
| Target gene | Sequences |
| Mouse Gapdh-F | 5′-TGGCCTTCCGTGTTCCTAC-3′ |
| Mouse Gapdh-R | 5′-GAGTTGCTGTTGAAGTCGCA-3′ |
| Mouse MyoD-F | 5′-CGGGACATAGACTTGACAGGC-3′ |
| Mouse MyoD-R | 5′-TCGAAACACGGGTCATCATAGA-3′ |
| Mouse Mettl3-F | 5′-CTGGGCACTTGGATTTAAGGAA-3′ |
| Mouse Mettl3-R | 5′-TGAGAGGTGGTGTAGCAACTT-3′ |
| Mouse Zc3h13-F | 5'-ATCCCGAAGACCTAGCGTATT-3' |
| Mouse Zc3h13-R | 5'-TGAAGGGCCATGTATGAACCT-3' |
| Mouse Virma-F | 5′-GGTTCGTTTTCCGTGTGTGG-3′ |
| Mouse Virma-R | 5′-GCCACTATGGGCTCGTACTC-3′ |
| Mouse Mettl4-F | 5′-GAGCTGAGAGTGCGGATAGC-3′ |
| Mouse Mettl4-R | 5′-GCAGATGTATCATAGGAAGCCC-3′ |
| Mouse Mettl16-F | 5′-CAAGGACAAACCACCTGACTT -3′ |
| Mouse Mettl16-R | 5′-GTGGGAATTAGTCTCTCCAAAGG-3′ |
| Mouse Wtap-F | 5′-ATGGCACGGGATGAGTTAATTC-3′ |
| Mouse Wtap-R | 5′- TTCCCTTAAACCAGTCACATCG-3′ |

| Table S2. Primer sequences for MeRIP-qPCR | |
| --- | --- |
| Target gene | Sequences |
| Mouse MyoD-seg1-F | 5′-CCCAGGACACGACTGCTTTCT-3′ |
| Mouse MyoD-seg1-R | 5′-ACTTTCTGCCACTCCGGAACCC-3′ |
| Mouse MyoD-seg2-F | 5′-CCTTTGTCATTGTACTGTTGGGGT-3′ |
| Mouse MyoD-seg2-R | 5′-GCGATAGAAGCTCCATATCCCAG-3′ |
| Mouse MyoD-seg3-F | 5′-ATGGAGCTTCTATCGCCGC-3′ |
| Mouse MyoD-seg3-R | 5′-TCGAAACACGGGTCATCATAGA-3′ |
| Mouse MyoD-seg4-F | 5′-ATGATGACCCGTGTTTCGACT-3′ |
| Mouse MyoD-seg4-R | 5′-CACCGCAGTAGGGAAGTGT-3′ |
| Mouse MyoD-seg5-F | 5′-CTCAAGCGCTGCACGTCCAG-3′ |
| Mouse MyoD-seg5-R | 5′-TGTAGTGCTCGCTGCCACGG-3′ |
| Mouse MyoD-seg6-F | 5′-GGCTGTGTCGAGCCTCGACT-3′ |
| Mouse MyoD-seg6-R | 5′-CATTGGGGTTTGAGCCTGCA-3′ |

Figure S1


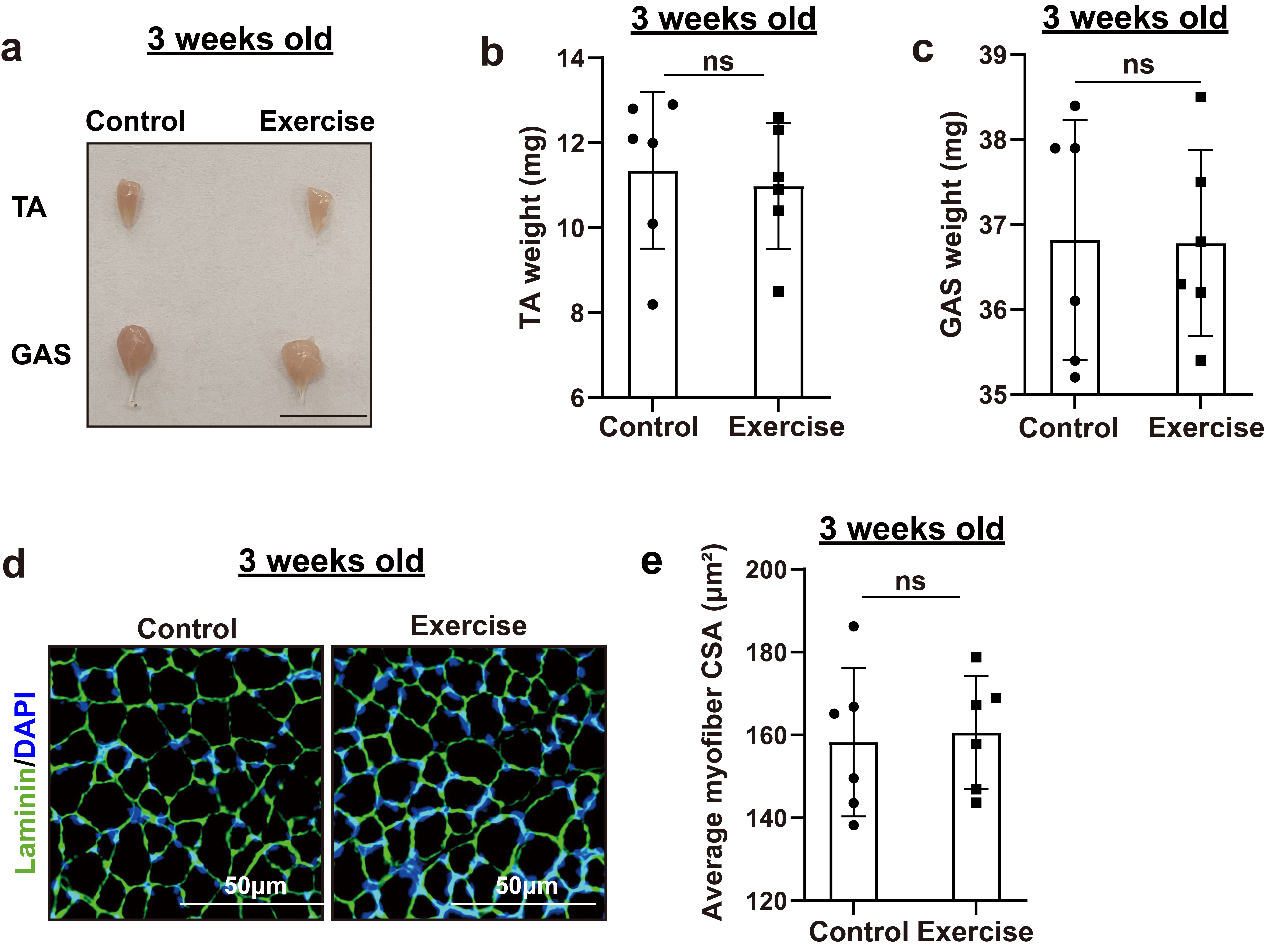


Fig. S1 The baseline data of skeletal muscle in 3 weeks old mice.

a. Gross appearance of tibialis anterior (TA) muscle and gastrocnemius (GAS) muscle for 3-week-old male mice in control and exercise groups (n=6). Scale bar, 1 cm.

b, c. Statistical analysis for the weight of tibialis anterior (TA) muscle and gastrocnemius (GAS) muscle for 3-week-old male mice in control and exercise groups (n=6).

d. Immunofluorescence staining of Laminin (green) in tibialis anterior (TA) muscle for 3-week-old male mice in control and exercise groups. Scale bar, 50 μm.

e. Statistical analysis of the average cross-sectional area (CSA) of myofibers in tibialis anterior (TA) muscle for 3-week-old male mice in control and exercise groups (n=6).

Data were presented as mean ± SD. ns indicated no significant changes.

Figure S2





Fig. S2 Exercise contributed to increased expression of Mettl3 and MyoD of MuSCs in adolescents.

1. The relative mRNA expression of *Mettl14*, *Mettl16* and *Wtap* in the tibialis anterior (TA) muscle between control and exercise mice (n=3).
2. The relative mRNA expression of *Mettl14*, *Mettl16* and *Wtap* for MuSCs isolated from control (CON-MuSCs) and exercise mice (EXE-MuSCs) (n=3).

c, d. Immunofluorescence staining of Mettl3 (green) and statistical analysis of fluorescence intensity for MuSCs isolated from control (CON-MuSCs) and exercise mice (EXE-MuSCs) (n=3). Scale bar, 50 μm.

e, f. Immunofluorescence staining and quantification analysis of MyoD^+^ cells for MuSCs isolated from control (CON-MuSCs) and exercise mice (EXE-MuSCs) (n=3). Scale bar, 50 μm.

Data were presented as mean ± SD. * indicated P < 0.05, ** indicated P < 0.01, *** indicated P < 0.001, ns indicated no significant changes.

Figure S3


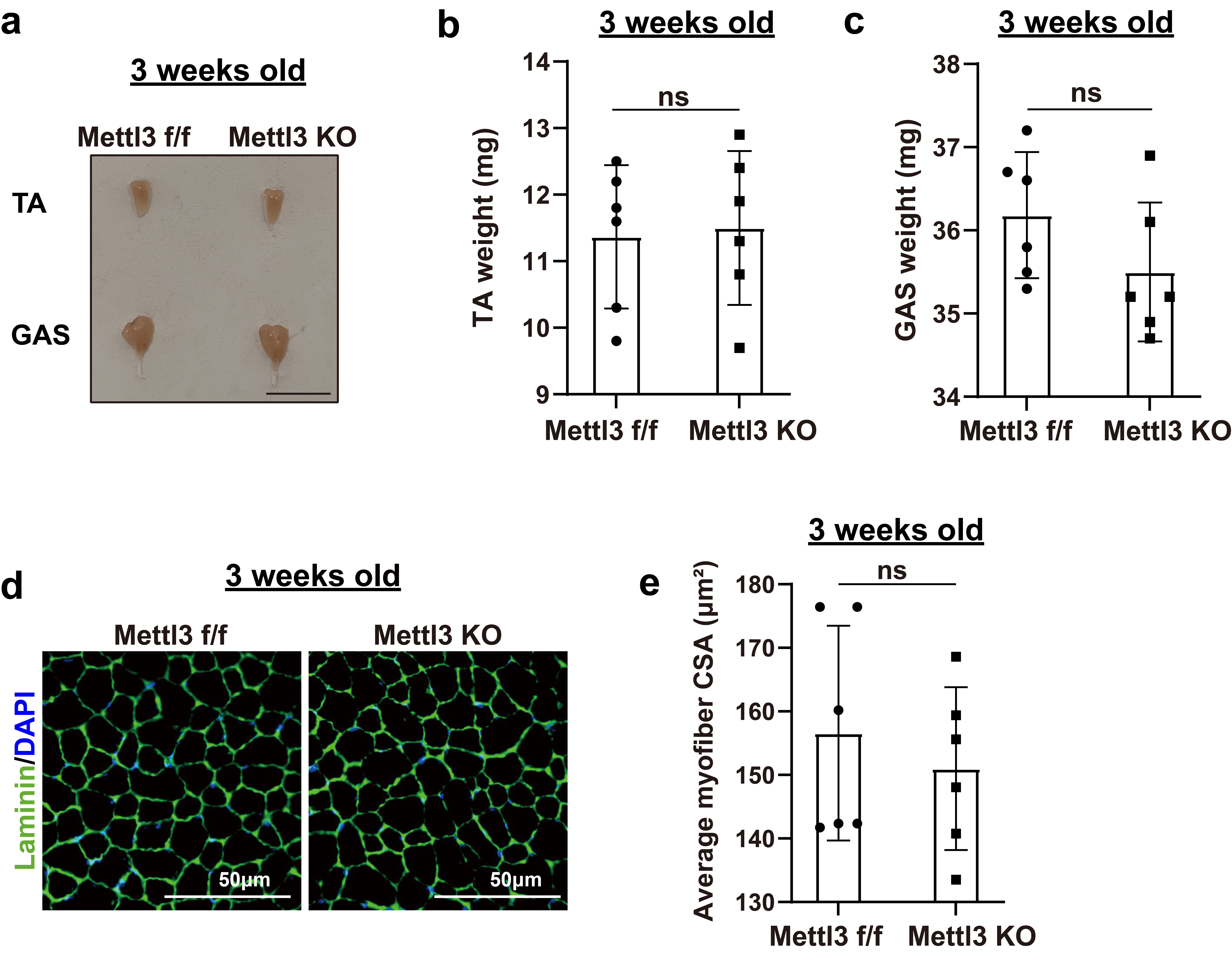


Fig. S3 The baseline skeletal muscle data of MuSC-specific *Mettl3^f/f^* and *Mettl3* KO mice at 3 weeks old.

1. Gross appearance of tibialis anterior (TA) muscle and gastrocnemius (GAS) muscle of MuSC-specific *Mettl3^f/f^* and *Mettl3* KO mice at 3 weeks old. Scale bar, 1 cm.

b, c. Statistical analysis for the weight of tibialis anterior (TA) muscle and gastrocnemius (GAS) muscle of MuSC-specific *Mettl3^f/f^* and *Mettl3* KO mice at 3 weeks old (n=6).

d. Immunofluorescence staining of Laminin (green) in tibialis anterior (TA) muscle from MuSC-specific *Mettl3^f/f^* and *Mettl3* KO mice at 3 weeks old. Scale bar, 50 μm.

e. Statistical analysis of the average cross-sectional area (CSA) of myofibers in tibialis anterior (TA) muscle from MuSC-specific *Mettl3^f/f^* and *Mettl3* KO mice at 3 weeks old (n=6).

Data were presented as mean ± SD. ns indicated no significant changes.

Figure S4


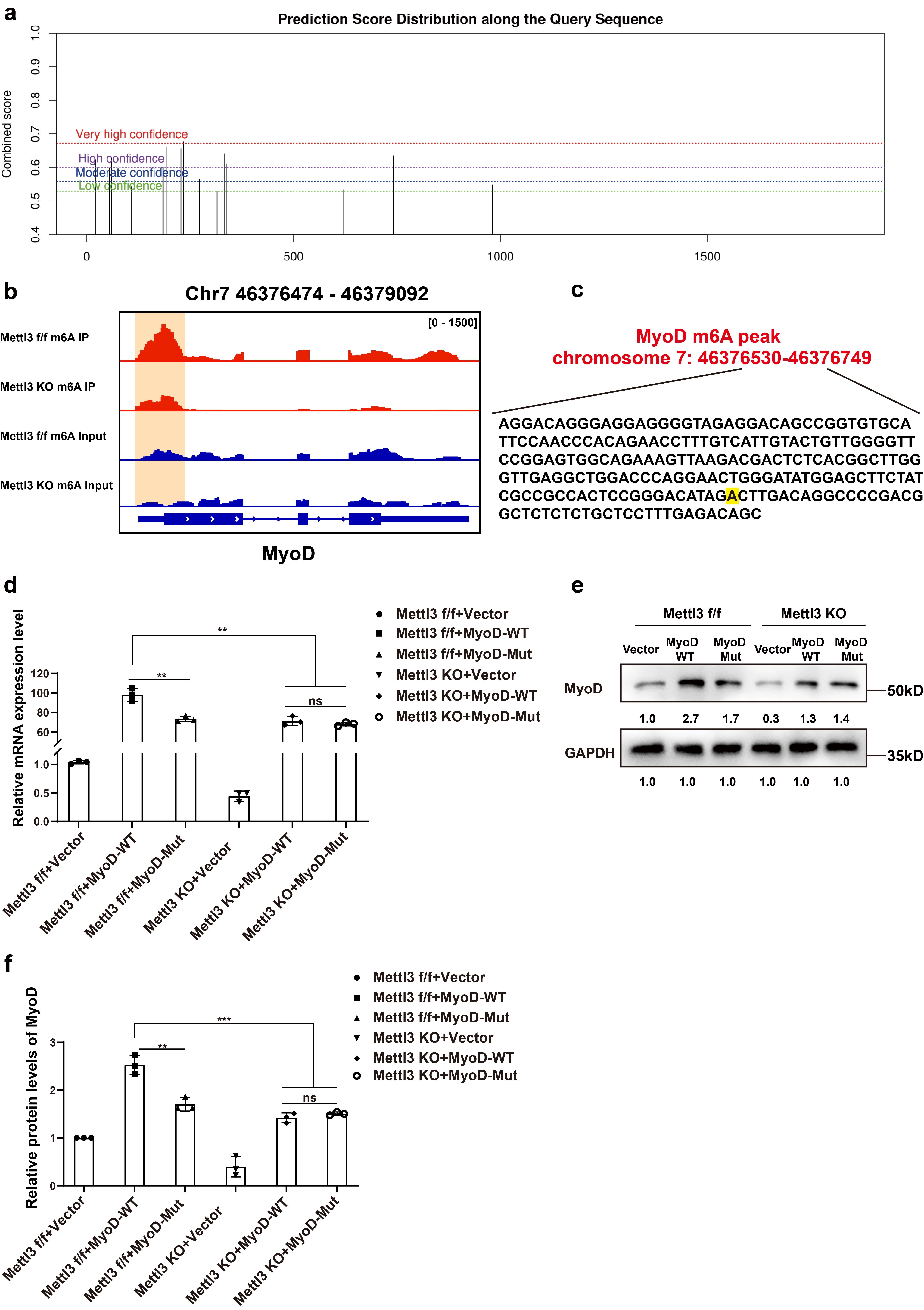


**Fig. S4 Mettl3-mediated m6A methylation regulated the stability of *MyoD* mRNA at the post-transcriptional level in MuSCs.**

1. The potential sites of m6A methylation in MyoD (NM_010866.2) mRNA which were predicted by SRAMP.
2. The m6A peak visulization of MeRIP-Seq in MyoD transcripts in *Mettl3^f/f^* and *Mettl3* KO MuSCs.

c. Detailed gene sequence about the m6A peak of MyoD located at chromosome 7: 46376530-46376749. The verified functional m6a modification site 234bp A (highlighted) was also located in this gene sequence.

d. The relative mRNA expression of *MyoD* in *Mettl3^f/f^* and *Mettl3* KO MuSCs after transfected with pcDNA-MyoD-WT or pcDNA-MyoD-Mut for 24 hours (n=3).

e, f. Western blot and qualification analysis of relative protein expression of MyoD and GAPDH in *Mettl3^f/f^* and *Mettl3* KO MuSCs after transfected with pcDNA-MyoD-WT or pcDNA-MyoD-Mut for 48 h (n=3).

Data were presented as mean ± SD. * indicated P < 0.05, ** indicated P < 0.01, *** indicated P < 0.001, ns indicated no significant changes.
